# Orthostatic hypotension without co-existent supine hypertension is associated with impaired cerebral oxygenation: findings from the Irish Longitudinal Study on Ageing (TILDA)

**DOI:** 10.1038/s41371-026-01125-w

**Published:** 2026-04-25

**Authors:** Louise Newman, John D. O’Connor, Richard B. Reilly, Rose Anne Kenny

**Affiliations:** 1 The Irish Longitudinal Study on Ageing, Trinity College Dublin, Dublin 2, Ireland; 2 School of Engineering, Ulster University, York Street, Belfast BT15 1AP, Co. Antrim, Northern Ireland UK; 3 Trinity Centre for Biomedical Engineering, Trinity College Dublin, Dublin 2, Ireland; 4 Discipline of Medical Gerontology, School of Medicine, Trinity College Dublin, Dublin, Ireland; 5 Mercer’s Institute for Successful Ageing, St James’s Hospital, Dublin, Ireland

**Keywords:** Risk factors, Ageing, Hypertension

## Abstract

Hypertension and orthostatic hypotension (OH) are common in older age, with both conditions recognized as risk factors for cardiovascular disease and end organ damage. Cerebral hypoperfusion is postulated to play a role in these adverse effects. For those with both hypertension and OH the effects on the cerebrovasculature are unclear, but there may be a risk of a higher hypotensive burden. We measured cerebral oxygenation utilizing near infrared spectroscopy, during an active stand challenge, and continuous blood pressure (BP), in The Irish Longitudinal Study on Ageing (TILDA) population. There was no difference in baseline oxygenation with supine hypertension (SH) or OH, but those with SH-OH had a higher supine BP, increased arterial stiffness and more cardiovascular conditions. Participants with SH-OH exhibited the largest BP drop and most impaired BP recovery on standing, yet the oxygenation response was not different to those with no SH and no OH. Those with OH only had the lowest BP values, lowest oxygenation values and most impaired oxygenation recovery, suggesting this group are at risk of cerebral hypotension when BP drops to low absolute values, whereas if BP is maintained at higher values as in those with SH-OH sufficient cerebral flow may be maintained.

## Introduction

Hypertension is common in older age, affecting more than 60% of those aged 60 years and older [1]. Orthostatic hypotension (OH) is also more common in older age, with up to 30% or more of older adults affected, depending on the population and setting [2–4]. Therefore, despite being paradoxical blood pressure (BP) states OH may co-exist with hypertension [5], especially in older adults, creating a treatment challenge for clinicians [6]. Hypertension is a risk factor for cardiovascular disease, end organ damage [7, 8] and cognitive decline [9]. OH is associated with adverse outcomes such as falls [10], cognitive impairment [11], depression [12] as well as also being an independent risk factor for cardiovascular disease and mortality [13]. Cerebral hypoperfusion is a posited pathway for these associations.

Furthermore, supine hypertension (SH) in those with OH is associated with an increased risk of adverse outcomes such as decreased renal function and increased white matter hyperintensities [14], as well as being associated with an increased risk of future falls [15]. There is also evidence of greater cognitive decline in those with coexistent SH and OH compared to those with only SH only [11, 16], suggesting that those with hypertension and OH may be at risk of a higher hypotensive burden.

Treatment of those with SH and OH is complex, and the treatment of OH may be prioritized if symptoms are a concern and affecting quality of life, but longer-term effects also need to be considered e.g., end organ damage. SH and the associated nocturnal non-dipping of BP is an independent risk factor for increased risk of falls due to exaggerated morning OH, as well as cardiovascular and cerebrovascular harm [17]. There are also concerns that lowering BP may increase the risk of OH and falls, or affect cerebral autoregulation and perfusion, especially in cohorts which have been excluded from larger BP treatment trials, such as those who are older, frailer or have comorbidities [18, 19].

The implications of coexistent hypertension and OH on the cerebrovasculature have yet to be elucidated, but it is important to investigate further in the context of challenging treatment decisions in this cohort [20]. The Irish Longitudinal Study on Ageing (TILDA) offers the opportunity to explore this pathway in a large population, with the introduction of near infrared spectroscopy (NIRS) monitoring alongside continuous BP monitoring during an active stand challenge.

The aim of this study was to assess the relationship between orthostatic hypotension and cerebral oxygenation, in those with and without SH. We hypothesized that those with SH-OH would have a higher cerebral hypotensive burden. This was assessed utilizing NIRS technology during an active stand challenge.

## Methods

### Sample

We analysed data from Wave 3 (2014–2015) of The Irish Longitudinal study on Ageing (TILDA), a nationally representative prospective cohort study of community-dwelling adults in the Republic of Ireland aged ≥50 years. The large cohort study collect data on health, lifestyle, social and financial circumstances of participants. Details of the study design and sampling criteria have been described previously [21, 22]. Briefly, sampling was based on random selections within geographical clusters. Data collection consists of computer-assisted interviews in the home as well as self-completion questionnaires. Participants are also invited to a comprehensive health assessment at a dedicated health centre. Those who attended the health centre and performed an active stand at Wave 3, and who had a normal BP reading in the seated position were included in this study. Ethical approval was provided by the Research Ethics Committee at Trinity College Dublin. The study adhered to the Declaration of Helsinki. All participants gave written informed consent. Those with a doctor’s diagnosis of Alzheimer’s, dementia or Parkinson’s disease were excluded.

### Active stand protocol

Participants lay supine for ~10 min in a quiet, ambient temperature room before standing as quickly as possible, assisted by a nurse where necessary. They remained standing for a further 3 min. A NIRS Portalite system (Artinis Medical Systems, Zetten, Netherlands) continuously monitored cerebral oxygenation from a single probe, at a rate of 50 Hz. This probe was affixed at approximately the left frontal FP1 position of the 10–20 electrode placement system [23]. Based on principles of adsorption and the modified Beer-Lambert law [24] concentrations of oxygenated hemoglobin (O2Hb) and deoxygenated hemoglobin (HHb) were determined using two wavelengths (nominally 760 nm and 850 nm). Tissue saturation index (TSI), a measure of absolute cerebral oxygenation, was calculated via spatial resolved spectroscopy using multiple transmitters, which had and inter-optode distances of 30 mm, 35 mm and 40 mm. This allowed for measurements at a maximum depth of 20 mm.

$${TSI}=\frac{O2{Hb}}{O2{Hb}+{HHb}}$$

A Finometer (Finapres Medical Systems, Arnhem, Netherlands) simultaneously recorded continuous systolic BP (SBP), diastolic BP (DBP) and heart rate utilizing digital photoplethysmography, sampled at 200 Hz. Multiple manual markers were applied throughout the test to synchronize the Finometer and NIRS data.

### Signal processing

Processing was implemented in MATLAB 9.7.0 (R2019b) Update 4 [25]. Finometer derived beat-to-beat data was interpolated to obtain equidistant datapoints at a rate of 1 Hz. NIRS data was downsampled to 1 Hz. Smoothing was applied via 10 s moving average filter ( ± 5 s) and an 11-point median filter. Baseline values were determined as the average of data between 60 and 30 s prior to onset of the stand. An algorithm utilizing data from the Finometer height correction unit [26] calculated the onset of the stand, as well the standing speed (transition time from supine to standing).

### Hypertension

#### Supine

Participants were classified as having SH if baseline supine SBP ≥ 140 mmHg and/or DBP ≥ 90 mmHg [27].

#### Seated

Two seated BP readings were taken one minute apart using an Omron™ digital oscillometric BP monitor (Omron M10-IT, Omron Inc. Kyoto, Japan) and the mean calculated to determine seated hypertension using the same BP cut-off points.

### Orthostatic hypotension

Based on previous normative data which demonstrated that non-recovery of BP by 30 s can be considered abnormal and thus an impaired response [4], OH40 was defined as a drop in SBP ≥ 20 mmHg and/or a drop in DBP ≥ 10 mmHg compared to baseline values, at 40 s after standing. We also used an alternative definition which adjusted for the presence of SH, whereby OH40 was defined as a drop in SBP ≥ 30 mmHg and/or a drop in DBP ≥ 15 mmHg compared to baseline values, at 40 s after standing [28].

### Covariates

Chosen covariates included age, sex, educational attainment, smoking history and problematic alcohol intake based on the CAGE questionnaire [29]. Health conditions included self-reported doctor’s diagnosis of diabetes, number of cardiovascular conditions (transient ischemic attack (TIA), stroke, hypertension, angina, heart attack, congestive heart failure (CHF), high cholesterol, heart murmur or any abnormal heart rhythm), cancer or kidney disease. Medication use included anti-hypertensives (ATC codes C02, C03, C07, C08 or C09), anti-depressants (ATC code N06A), benzodiazepines (ATC code N05BA) and anti-psychotics (ATC code N05A). Other selected measurements included height, body mass index (BMI), the timed-up-and-go (TUG) test (time taken to stand up from a chair, walk 3 m, turn and sit back down), depressive symptoms (short-form of the Centre for Epidemiological Studies Depression (CES-D) scale [30], standing speed, and pulse wave velocity (PWV) as a measure of arterial stiffness. Carotid-femoral PWV was determined using a Vicorder (Skidmore Medical Ltd, Bristol, UK) and calculated as an average of two readings.

### Statistical modelling

Statistical analysis was performed using Stata 15 (StataCorp, TX, USA). Histograms and Q-Q plots were used to assess the normality of the data. Differences in group characteristics for normally distributed continuous variables were assessed via an ANOVA. Mann-Whitney U tests were used for non-normal distributions, whilst categorical variables were compared using the Chi-square test.

Logistic regression was employed to assess the relationship between SH and OH. Model 1 was a univariate. Model 2 was minimally adjusted for age, sex and education only. Model 3 was adjusted for all covariates.

Linear multi-level mixed-effects models were then utilized to assess the BP, heart rate and cerebral response during the active stand in those with SH-OH. This analysis included active stand data at 10 s intervals from time 0–180 s. Time 0 s indicated the onset of the stand. Repeated measurements within participants were accounted for by fixed and random effects (random intercept). Linear splines were fitted to SBP and TSI signals with knots located at 0, 10, 20, 30, 40, 50 and 180 s thus reparametrizing data to phases of the response (0–10, 10–20, 20–30, 30–50 and 50–180 s), as described previously [31]. As points closer in time are more strongly correlated an autoregression covariance matrix with a lag of 1 was applied to model residual variance. We allowed for the effect of covariates to differ at each time phase by including an interaction for each time phase with each covariate.

Initially, we assessed the BP and heart rate response (absolute and change from baseline), adjusting for age, sex, education and height, by presence of SH and OH. Then we assessed the cerebral response (TSI, absolute and change from baseline) by presence of SH and OH. We performed three models when examining the TSI response. Model 1 was a univariate model. Model 2 adjusted for age, sex, education and height. A third model additionally adjusted for smoking history, alcohol excess, stand transition time, depressive symptoms, antidepressant medications, antihypertensive medications, benzodiazepines, antipsychotics, diabetes, number of cardiovascular conditions, cancer, kidney, disease, body mass index, TUG time, and PWV. All models were repeated utilizing the alternative definition of OH which adjusted for the presence of SH.

## Results

### Sample characteristics

**Fig. 1:**
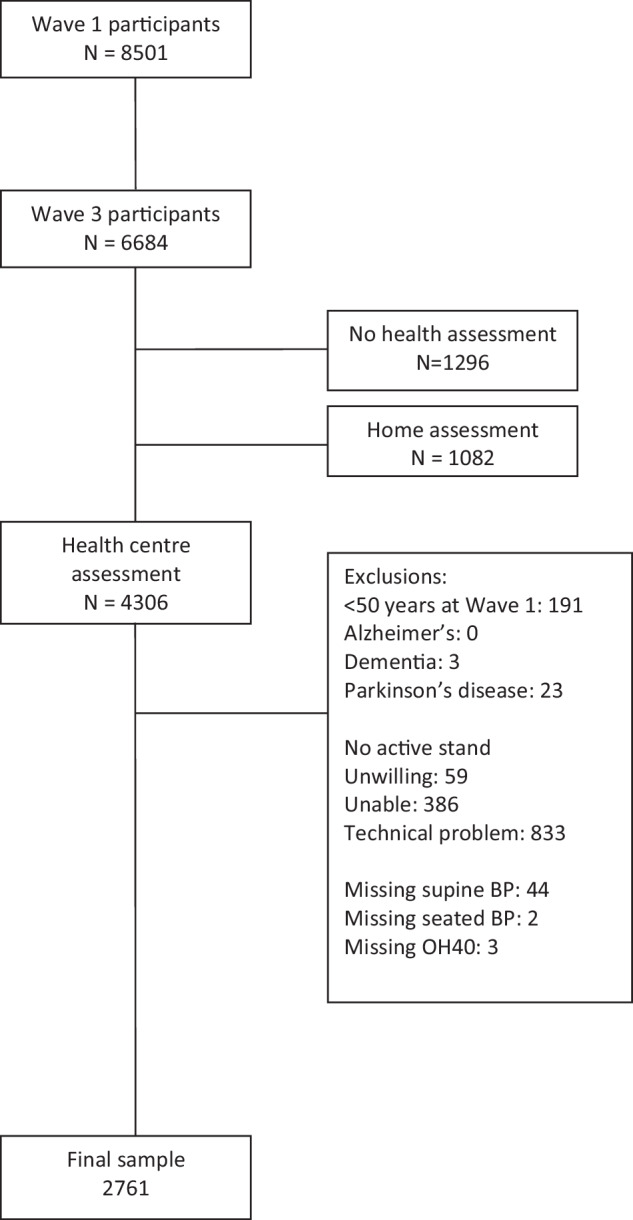
. Sample selected for analysis.

The final analysis sample consisted of 2,761 participants, fig. 1. Mean age of the sample was 64.9 (7.5) years, ranging from 50–93 years. The logistic regression demonstrated that those with SH were more likely to experience OH (univariate model: OR = 1.86, p = <0.001, 95% CI: 1.48–2.34; fully adjusted model: OR = 1.58, p = <0.001, 95% CI: 1.23–2.03). Characteristics by SH and OH status are reported in Table 1. Those with SH made up almost half the sample (49%). Of those who had SH 16% had OH (N = 224). Using the alternative definition of OH which adjusted for the presence of SH (Supplementary Table 1) the percentage of those with SH who also had OH reduced from 16–6.5% (N = 88). The characteristics of the four groups did not alter significantly with the adjusted definition, although the percentage of those with SH-OH who were taking anti-hypertensive medications rose to 58% versus 39% (from 52% versus 38%) in those with SH and no OH. Those with SH-OH were also more likely to be older, have a higher supine SBP and higher arterial stiffness, more CVD conditions, and slower TUG time. Baseline TSI was not different in any of the groups.

**Table 1:** Participant characteristic by supine hypertension and orthostatic hypotension status.

	No supine hypertension		Supine hypertension		Statistical test^
	No OH	Has OH	No OH	Has OH	p-value
	N 1266	N 134	N 1137	N 224	
Age (years), mean (SD)	63.2 (6.7)	67.8 (8.0)	65.7 (7.3)	69.0 (8.2)	<0.001*
Sex (female), n (%)	596 (47)	56 (42)	670 (59)	123 (55)	<0.001*
Highest educational attainment					
Primary/none, n (%)	187 (15)	29 (22)	196 (17)	46 (21)	
Secondary, n (%)	514 (41)	54 (40)	459 (40)	86 (38)	0.172
Tertiary, n (%)	565 (45)	51 (38)	482 (42)	92 (41)	
Height (cm), mean (SD)	167.6 (9.1)	169.0 (9.2)	164.7 (9.0)	165.4 (9.1)	<0.001*
Seated SBP (mmHg), mean (SD)	124.8 (15.0)	124.0 (16.1)	141.3 (17.2)	141.2 (17.6)	<0.001*
Seated DBP (mmHg), mean (SD)	77.5 (8.8)	74.9 (8.2)	84.9 (10.3)	83.0 (10.5)	<0.001*
Seated heart rate (bpm), mean (SD)	68.5 (11.2)	67.1 (11.1)	68.5 (10.8)	68.4 (10.3)	0.992
Supine SBP (mmHg), mean (SD)	124.9 (11.0)	125.9 (10.7)	157.2 (14.9)	163.5 (19.3)	<0.001*
Supine DBP (mmHg), mean (SD)	70.6 (7.1)	71.9 (6.9)	81.3 (8.9)	83.5 (10.5)	<0.001*
Supine heart rate (bpm), mean (SD)	65.0 (10.0)	64.6 (9.5)	65.6 (9.5)	65.9 (9.5)	0.076
Supine TSI (%), mean (SD)	72.7 (4.7)	72.2 (4.9)	72.6 (5.1)	72.8 (5.0)	0.759
PWV (m/sec), mean (SD)	10.0 (1.9)	10.9 (2.2)	10.7 (2.0)	11.4 (2.2)	<0.001*
BMI, mean (SD)	28.5 (4.8)	27.9 (4.8)	28.4 (4.4)	27.2 (4.6)	0.038*
Smoking history					
Never	612 (48)	55 (41)	544 (48)	115 (51)	
Past	530 (42)	64 (48)	492 (43)	90 (40)	0.606
Current	124 (10)	15 (11)	101 (9)	19 (9)	
Alcohol excess, n (%)					
No (CAGE < 2)	980 (77)	100 (75)	871 (76)	181 (81)	
Yes (CAGE > = 2)	156 (12)	12 (9)	144 (13)	29 (13)	0.107
Did not respond	130 (10)	22 (16)	122 (11)	14 (6)	
Diabetes, n (%)	88 (7)	15 (11)	60 (5)	21 (9)	0.013
CVD conditions					
= 0, n (%)	560 (44)	48 (36)	413 (36)	66 (29)	
= 1, n (%)	452 (36)	45 (33)	419 (37)	86 (38)	<0.001*
>= 2, n (%)	254 (20)	41 (31)	305 (27)	72 (32)	
Cancer, n (%)	34 (3)	7 (5)	34 (3)	7 (3)	0.434
Kidney disease, n (%)	6 (1)	1 (1)	5 (1)	2 (1)	0.815
Taking antihypertensive medications, n (%)	417 (33)	67 (50)	432 (38)	117 (52)	<0.001*
Taking antidepressant medications, n (%)	93 (7)	14 (10)	72 (6)	15 (7)	0.324
Taking antipsychotic medications, n (%)	11 (1)	4 (3)	18 (2)	1 (1)	0.070
Taking benzodiazepines, n (%)	11 (1)	2 (1)	15 (1)	2 (1)	0.702
CES-D score (max = 22), mean (SD)	3.8 (3.7)	3.6 (3.8)	3.8 (3.6)	4.1 (3.8)	0.522
TUG (s), mean (SD)	8.9 (2.2)	9.3 (2.2)	9.0 (1.8)	9.5 (1.9)	<0.001*
Stand time transition (s), mean (SD)	7.2 (2.8)	7.6 (2.9)	7.2 (2.7)	7.8 (3.5)	0.110
MOCA, median (IQR)	27 (25–29)	26 (23 – 28)	26 (24–28)	26 (24–28)	<0.001*

Total sample N = 2761.

*CES-D* centre for epidemiological studies depression scale (short form), *IQR* interquartile range, *MOCA* montreal cognitive assessment, *TUG* timed up and go.

*p < 0.05.

### SH and OH

#### BP response

**Fig. 2:**
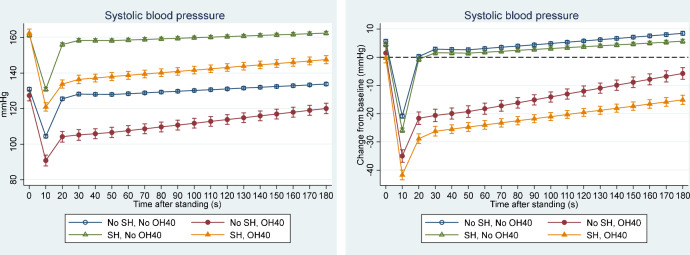
Systolic blood pressure response, adjusted for age, sex, education and height, conditional means and 95% confidence intervals from mixed-effects models. Absolute values (left) and change from baseline (right) are shown. SH – supine hypertension, OH40 – OH at 40 s post standing. Supine baseline level denoted by black dashed line.

Those with SH maintained higher absolute values of SBP throughout the stand (with or without OH), fig. 2 (left). The group with OH, but no SH had the lowest absolute SBP values throughout the stand, dropping to 90.8 mmHg [95% confidence intervals (CI): 87.8 to 93.9] at 10 s. Those with SH-OH experienced the largest drop in SBP from baseline (−41.7 mmHg [95% CI: −43.5–−40.0]) and exhibited the poorest recovery, fig. 2 (right). However, the minimum SBP value in this group only dropped to 121.0 mmHg [95% CI: 118.7 to 123.4]. Those with no SH and no OH recovered to baseline SBP levels within 20 s, whilst those with OH remained below baseline levels after three minutes (SH-OH group at 180 s: −15.1 mmHg [95% CI: −16.7–−13.5]; OH no SH group: −5.7 mmHg [95% CI: −7.8–−3.6]). A similar pattern was observed in the absolute DBP response (see Supplementary fig. 1, left). Examining the change in DBP from baseline we observed no difference in those groups with or without SH, but there was a much larger drop in those with OH and they had the most impaired recovery (Supplementary fig. 1, right).

Heart rate is shown in Supplementary fig. 2. There were no differences in increases in HR between the groups. However, the SH-OH stabilized at a level closest to the baseline supine HR, whereas the group with OH but no SH stabilized at the highest level above the supine baseline level.

A similar pattern was observed when the definition of OH was adjusted for the presence of SH, as per Supplementary figs. 3–5.

#### Cerebral oxygenation response

**Fig. 3:**
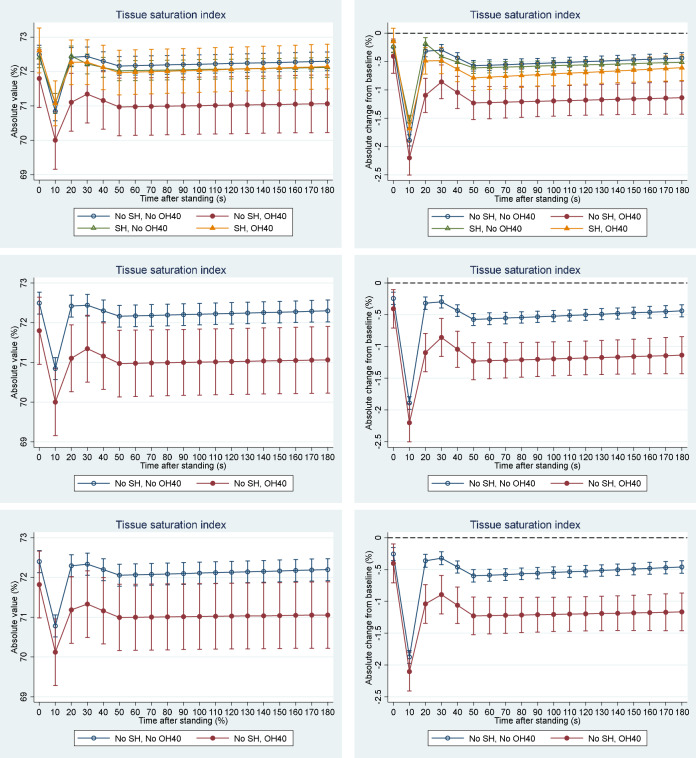
Tissue saturation index response, conditional means with 95% confidence intervals from mixed-effects models. Absolute values (left) and change from baseline (right) are shown. The top panel shows the unadjusted model for all groups. The middle panel shows the statistically different groups only (unadjusted model). The bottom panel shows the fully adjusted model (statistically different groups only). SH – supine hypertension, OH40 – OH at 40 s post standing. Supine baseline level denoted by black dashed line.

There were no differences in baseline TSI between the groups. Those with OH but no SH experienced the lowest absolute values of TSI throughout the stand recovery from 20 s onwards, fig. 3 (left). This group also maintained the largest difference from baseline TSI, from 20 s through to 180 s (Fully adjusted model: −1.04% [95% CI: −1.35–−0.74] at 20 s and −1.17% [95% CI: −1.46–−0.87] at 180 s), fig. 3 (right). There were no differences in the initial drop between groups. Controlling for the minimal and then full set of covariates made a modest difference to the SH-OH group when examining the absolute TSI values. There were no significant differences between the other (no OH) groups. All groups stabilized at levels below supine TSI values. A similar pattern was observed when the definition of OH was adjusted for the presence of SH, as per Supplementary fig. 6.

## Discussion

This study examined both blood pressure and cerebral flow in a cohort with and without supine hypertension and/or orthostatic hypotension. Those with SH-OH experienced the largest BP drop and most impaired BP recovery on standing. Despite these BP changes the TSI response in those with SH-OH was not different to the group with no SH and no OH, and there was no sustained cerebral hypotension in those with SH-OH. However, our data demonstrated that those with OH but no SH are at risk of cerebral hypotension. They had the lowest absolute BP values and TSI values during the active stand, and experienced the most impaired TSI recovery. These findings indicate that the absolute BP value may be more important in the context of OH and maintaining cerebral blood flow (CBF), and may also suggest that the cerebral autoregulatory system adapts to the higher BP in those with SH.

Contrary to our hypothesis we did not find evidence of a higher hypotensive load in those with SH-OH, despite indications of more advanced atherosclerosis in this group (higher SBP, higher PWV, more CVD conditions). In those with OH and no SH, SBP dropped to ~90 mmHg upon standing (delta SBP = ~35 mmHg), whereas SBP only dropped to ~120 mmHg in those with SH-OH despite this group experiencing the largest drop from baseline (~42 mmHg). This suggests that for a large drop in BP, but where the absolute value remains at a reasonable or normal value or above a threshold value, CBF can recover quickly after a large perturbation. This may imply that the treatment of SH-OH may be safer than some contend. Therefore, more aggressive therapy may overall be beneficial for those with SH-OH. The observation aligns with our recent meta-analysis where we showed that the exclusion of patients with OH from randomised controlled trials does not appear to affect the relative risk estimates for falls and syncope in antihypertensive trials [32].

However, in this study after a drop in SBP to a low absolute SBP value (in those with OH and no SH) autoregulation was less able to restore the drop in cerebral perfusion as TSI remained more than 1% lower than supine baseline levels in this group even in the stabilization period. This highlights the need to monitor the full dynamic response to an active stand if those with SH-OH are to be treated with anti-hypertensives. A cerebral oxygenation deficit similar to the magnitude in this study has previously been shown to be clinically relevant as it is associated with outcomes such as late life depression [33], and cerebral oxygenation deficits around 2 to 10% are associated with syncope in tilt-table tests [34]. All groups stabilized at values lower than baseline, which is a consistent finding in NIRS studies examining supine to standing postural change [35].

A clinical concern in those with SH-OH is that intensively reducing BP may either lead to reduced cerebral perfusion, or increased episodes of OH and subsequently related adverse events. However, some small studies suggests that CBF is maintained or even increased [36, 37], and that cerebral autoregulation is preserved [38–40] after BP lowering.

An expanded autoregulatory range in those with OH may be one explanation for the preserved cerebral oxygenation levels reported in this study. Some patients with severe autonomic dysfunction can tolerate large changes in BP [41]. However, this is generally observed in patients with neurogenic OH, and this community-dwelling cohort of older adults, do not have adrenergic failure or neurogenic OH but rather age-related failure to stabilize orthostatic changes in blood pressure within the 40 s time frame.

Results from the Systolic Blood Pressure Intervention Trial (SPRINT) reported a decrease in the risk of OH with BP lowering, but the rate of hypotension and syncope were higher in the intensive treatment group versus the standard treatment group, although injurious falls were lower [42]. However, the exclusion criteria in SPRINT included diabetes, history of stroke and other comorbidities, and therefore there are concerns on the applicability of the results to older, frailer cohorts with comorbidities [18]. SPRINT also utilized a sit-to-stand protocol to determine OH status as opposed to the more physiologically challenging supine-to-stand, underestimating OH rates. They also measured OH after one minute of standing, failing to capture early deficits in BP which predict future falls. SPRINT did not include an objective measure of cerebral hemodynamics during the postural challenge but depended on discrete BP measurements and adverse outcomes only to assess effects of BP lowering. Although more recent analysis in an magnetic resonance imaging sub-study utilizing arterial spin labelling to quantify CBF (n = 315, 3% of the original cohort; 4-year follow-up) suggests that CBF may be increased with intensive BP lowering [43].

For future therapeutic trials it would be pertinent to include an objective measure of cerebral hemodynamics, and ideally continuous noninvasive BP monitoring to detect the full pattern of BP dynamics during any postural challenges. Longitudinal studies with repeated cerebral hemodynamic measures would assist in gaining further insight on the relationships.

We have previously shown that cerebral oxygenation recovery on standing is impaired in those taking anti-hypertensives [44], thus included it as a covariate in the present study and therefore our findings are independent of anti-hypertensive use. We also controlled for PWV as a measure of arterial stiffness as well as TUG time as a measure of sarcopenia and muscle strength, but this did not alter our findings.

In another previous study we showed that cerebral oxygenation response to orthostasis is impaired if adjusted for SBP during the stand [45], implying the autoregulatory system has adapted to the higher BP, which is again demonstrated here in this study. This is in agreement with the studies that find no difference in cerebral autoregulation efficacy in hypertension [39], or CBF is unchanged [36], as well as studies which suggest cerebral autoregulation readapts to lower BP [46] post treatment.

### Strengths and limitations

Strengths of this study are that we used a large population cohort with a wide continuum of ages, and comorbidities. This included older frail participants with co-morbidities, lacking in other studies such as SPRINT, and we were then able to adjust for these as covariates in our models. Our study utilized continuous BP measures thus capturing rapid and complete dynamics of the BP response throughout the postural challenge. The postural challenge was a supine-to-stand which elicits a larger response than a sit-to-stand.

Our study included a mix of treated and untreated hypertension and did not account for duration of disease and treatment which may be important. Previously it has been shown that there are low levels of treatment and control of hypertension in this population cohort [47] therefore we relied on objective measures of hypertension and included anti-hypertensive use as a covariate to account for both of these confounding factors. Those who experienced the most severe symptoms upon standing and had to abandon the test were excluded as full data was not available, but they may also represent those who had the most impaired cerebral hemodynamic response.

## Conclusion

Those with OH are vulnerable to cerebral hypotension when absolute BP drops to low absolute values. Those with coexisting supine hypertension and OH experience large drops in BP but sufficient cerebral flow may be maintained as absolute BP values do not reach abnormally low values, as in the case of isolated OH. Future studies should include monitoring of cerebral hemodynamics, as monitoring peripheral BP is not sufficient, and does not directly translate to cerebral measures.

## Summary

### What is known about topic

- Cerebral hypoperfusion is hypothesized to play a role in the adverse effects related to hypertension and orthostatic hypotension (OH)
- Treating those with co-existing hypertension and OH is a clinical challenge

### What this study adds

:

- Provides evidence in a large population sample demonstrating cerebral oxygenation responses during an active stand challenge in those with hypertension and with/without OH.
- Participants with both hypertension and OH exhibited the largest BP drop and most impaired BP recovery on standing, yet the oxygenation response was not different to those with no hypertension and no OH.
- Those with OH only had the lowest BP values, lowest oxygenation values and most impaired oxygenation recovery suggesting this group are at risk of cerebral hypotension when BP drops to low absolute values, whereas if BP is maintained at higher values as in those with SH-OH sufficient cerebral flow may be maintained.

## Supplementary information

Supplemental data

## Data Availability

Researchers interested in using TILDA data may access the data for free from the following site: Irish Social Science Data Archive at University College Dublin http://www.ucd.ie/issda/data/tilda/. For access to more sensitive and detailed data, including the BP and NIRS data, an application may be made to the Hot Desk Facility via tilda.hotdesk@tcd.ie.

## References

[1] Chow CK, Teo KK, Rangarajan S, Islam S, Gupta R, Avezum A, et al. Prevalence, awareness, treatment, and control of hypertension in rural and urban communities in high-, middle-, and low-income countries. Jama. 2013;310:959–68. 24002282 10.1001/jama.2013.184182

[2] Rutan GH, Hermanson B, Bild DE, Kittner SJ, LaBaw F, Tell GS. Orthostatic hypotension in older adults. the cardiovascular health study. CHS collaborative research group. Hypertension. 1992;19:508–19. 1592445 10.1161/01.hyp.19.6.508

[3] Iwanczyk L, Weintraub NT, Rubenstein LZ. Orthostatic hypotension in the nursing home setting. J Am Med Dir Assoc. 2006;7:163–7. 16503309 10.1016/j.jamda.2005.10.011

[4] Finucane C, O’Connell MDL, Fan CW, Savva GM, Soraghan CJ, Nolan H, et al. Age-Related normative changes in phasic orthostatic blood pressure in a large population study findings from the irish longitudinal study on ageing (TILDA). Circulation. 2014;130:1780–9. 25278101 10.1161/CIRCULATIONAHA.114.009831

[5] Di Stefano C, Milazzo V, Totaro S, Sobrero G, Ravera A, Milan A, et al. Orthostatic hypotension in a cohort of hypertensive patients referring to a hypertension clinic. J Hum Hypertens. 2015;29:599–603. 25631221 10.1038/jhh.2014.130

[6] Chisholm P, Anpalahan M. Orthostatic hypotension: pathophysiology, assessment, treatment and the paradox of supine hypertension. Intern Med J. 2017;47:370–9. 27389479 10.1111/imj.13171

[7] Mancia G, Fagard R, Narkiewicz K, Redón J, Zanchetti A, Böhm M, et al. 2013 ESH/ESC guidelines for the management of arterial hypertension: the task force for the management of arterial hypertension of the European Society of Hypertension (ESH) and of the European Society of Cardiology (ESC). J Hypertension. 2013;31:1281–357. 10.1097/01.hjh.0000431740.32696.cc 23817082

[8] Hypertension EETFftMoA. 2013 Practice guidelines for the management of arterial hypertension of the European Society of Hypertension (ESH) and the European Society of Cardiology (ESC): ESH/ESC Task Force for the Management of Arterial Hypertension. J Hypertens. 2013;31:1925–38. 24107724 10.1097/HJH.0b013e328364ca4c

[9] Iadecola C, Yaffe K, Biller J, Bratzke LC, Faraci FM, Gorelick PB, et al. Impact of hypertension on cognitive function: a scientific statement from the american heart association. Hypertension. 2016;68:e67–e94. 27977393 10.1161/HYP.0000000000000053 PMC5361411

[10] Finucane C, O’Connell MD, Donoghue O, Richardson K, Savva GM, Kenny RA. Impaired orthostatic blood pressure recovery is associated with unexplained and injurious falls. J Am Geriatr Soc. 2017;65:474–82. 28295143 10.1111/jgs.14563

[11] McNicholas T, Tobin K, Carey D, O’Callaghan S, Kenny RA. Is baseline orthostatic hypotension associated with a decline in global cognitive performance at 4-year follow-up? data from TILDA (The Irish Longitudinal Study on Ageing). J Am Heart Assoc. 2018;7:e008976. 30371298 10.1161/JAHA.118.008976 PMC6404900

[12] Briggs R, Carey D, Kennelly SP, Kenny RA. Longitudinal association between orthostatic hypotension at 30 s post-standing and late-life depression. Hypertension. 2018;71:946–54. 29632103 10.1161/HYPERTENSIONAHA.117.10542

[13] Fedorowski A, Stavenow L, Hedblad B, Berglund G, Nilsson PM, Melander O. Orthostatic hypotension predicts all-cause mortality and coronary events in middle-aged individuals (The Malmo Preventive Project). Eur Heart J. 2010;31:85–91. 19696189 10.1093/eurheartj/ehp329 PMC2800919

[14] Palma JA, Redel-Traub G, Porciuncula A, Samaniego-Toro D, Millar Vernetti P, Lui YW, et al. The impact of supine hypertension on target organ damage and survival in patients with synucleinopathies and neurogenic orthostatic hypotension. Parkinsonism Relat Disord. 2020;75:97–104. 32516630 10.1016/j.parkreldis.2020.04.011 PMC7415666

[15] Donoghue OA, O’Connell MDL, Bourke R, Kenny RA. Is orthostatic hypotension and co-existing supine and seated hypertension associated with future falls in community-dwelling older adults? results from The Irish Longitudinal Study on Ageing (TILDA). PLOS ONE. 2021;16:e0252212. 34043698 10.1371/journal.pone.0252212 PMC8158994

[16] Frewen J, Finucane C, Savva GM, Boyle G, Kenny RA. Orthostatic hypotension is associated with lower cognitive performance in adults aged 50 plus with supine hypertension. J Gerontol: Ser A. 2014;69:878–85. 10.1093/gerona/glt171 24214492

[17] Yano Y, Kario K. Nocturnal blood pressure and cardiovascular disease: a review of recent advances. Hypertension Res. 2012;35:695–701. 10.1038/hr.2012.26 22378470

[18] Sexton DJ, Canney M, O’Connell ML, et al. Injurious falls and syncope in older community-dwelling adults meeting inclusion criteria for sprint. JAMA Intern Med. 2017;177:1385–7. 28715566 10.1001/jamainternmed.2017.2924 PMC5818831

[19] Anderson TS, Odden MC, Penko J, Kazi DS, Bellows BK, Bibbins‐Domingo K. Characteristics of populations excluded from clinical trials supporting intensive blood pressure control guidelines. J Am Heart Assoc. 2021;10:e019707. 33754796 10.1161/JAHA.120.019707 PMC8174340

[20] Juraschek SP, Cortez MM, Flack JM, Ghazi L, Kenny RA, Rahman M, et al. Orthostatic hypotension in adults with hypertension: a scientific statement from the american heart association. Hypertension. 2024;81:e16–e30. 38205630 10.1161/HYP.0000000000000236 PMC11067441

[21] Cronin H, O’Regan C, Finucane C, Kearney P, Kenny RA. Health and aging: development of the Irish longitudinal study on ageing health assessment. J Am Geriatr Soc. 2013;61:S269–78. 23662719 10.1111/jgs.12197

[22] Whelan BJ, Savva GM. Design and methodology of the irish longitudinal study on ageing. J Am Geriatr Soc. 2013;61:S265–8. 23662718 10.1111/jgs.12199

[23] Jasper HH. The ten twenty electrode system of the international federation. Electroencephalography Clin Neurophysiol. 1958;10:371–5. 10590970

[24] Ferrari M, Quaresima V. Near infrared brain and muscle oximetry: from the discovery to current applications. J Infrared Spectrosc. 2012;20:1–14.

[25] Mathworks. MATLAB version 9.7.0 (R2019b) Update 4 Natick, Massachusetts, United States: The MathWorks Inc.; 2019.

[26] O’Connor JD, O’Connell MDL, Nolan H, Newman L, Knight SP, Kenny RA. Impact of standing speed on the peripheral and central hemodynamic response to orthostasis. Hypertension. 2020;75:524–31. 31838912 10.1161/HYPERTENSIONAHA.119.14040

[27] Fanciuli A, Jordan J, Biaggioni I, Calandra-Buonaura G, Cheshire WP, Cortelli P, et al. Consensus statement on the definition of neurogenic supine hypertension in cardiovascular autonomic failure by the American Autonomic Society (AAS) and the European Federation of Autonomic Societies (EFAS) : Endorsed by the European Academy of Neurology (EAN) and the European Society of Hypertension (ESH). Clin Auton Res. 2018;28:355–62. 29766366 10.1007/s10286-018-0529-8 PMC6097730

[28] Gibbons CH, Schmidt P, Biaggioni I, Frazier-Mills C, Freeman R, Isaacson S, et al. The recommendations of a consensus panel for the screening, diagnosis, and treatment of neurogenic orthostatic hypotension and associated supine hypertension. J Neurol. 2017;264:1567–82. 28050656 10.1007/s00415-016-8375-x PMC5533816

[29] Bush B, Shaw S, Cleary P, Delbanco TL, Aronson MD. Screening for alcohol abuse using the CAGE questionnaire. Am J Med. 1987;82:231–5. 2880504 10.1016/0002-9343(87)90061-1

[30] O’Halloran AM, Kenny RA, King-Kallimanis BL. The latent factors of depression from the short forms of the CES-D are consistent, reliable and valid in community-living older adults. Eur Geriatric Med. 2014;5:97–102.

[31] O’Connell MD, Savva GM, Finucane C, Romero-Ortuno R, Fan CW, Kenny RA. Impairments in hemodynamic responses to orthostasis associated with frailty: results from the irish longitudinal study on ageing (TILDA). J Am Geriatrics Soc. 2018;66:1475–83. 10.1111/jgs.15327 29668044

[32] Reddin C, Murphy R, Hanrahan C, Loughlin E, Ferguson J, Judge C, et al. Randomised controlled trials of antihypertensive therapy: does exclusion of orthostatic hypotension alter treatment effect? a systematic review and meta-analysis. Age Ageing. 2023;52:afad044. 37014001 10.1093/ageing/afad044 PMC10883139

[33] Briggs R, Carey D, Claffey P, McNicholas T, Newman L, Nolan H, et al. The association between frontal lobe perfusion and depressive symptoms in later life. Br J Psychiatry. 2019;214:230–6. 30606275 10.1192/bjp.2018.288

[34] Bachus E, Holm H, Hamrefors V, Melander O, Sutton R, Magnusson M, et al. Monitoring of cerebral oximetry during head-up tilt test in adults with history of syncope and orthostatic intolerance. EP Europace. 2017;20:1535–1542. 10.1093/europace/eux298 29036615

[35] Klop M, de Heus RAA, Maier AB, van Alphen A, Floor-Westerdijk MJ, Bronkhorst M, et al. Capturing postural blood pressure dynamics with near-infrared spectroscopy-measured cerebral oxygenation. GeroScience. 2023;45:2643–57. 37041313 10.1007/s11357-023-00791-9 PMC10651596

[36] Tryambake D, He J, Firbank MJ, O’Brien JT, Blamire AM, Ford GA. Intensive blood pressure lowering increases cerebral blood flow in older subjects with hypertension. Hypertension. 2013;61:1309–15. 23529166 10.1161/HYPERTENSIONAHA.112.200972

[37] Lipsitz LA, Gagnon M, Vyas M, Iloputaife I, Kiely DK, Sorond F, et al. Antihypertensive therapy increases cerebral blood flow and carotid distensibility in hypertensive elderly subjects. Hypertension. 2005;45:216–21. 15655124 10.1161/01.HYP.0000153094.09615.11

[38] Lipsitz LA, Mukai S, Hamner J, Gagnon M, Babikian V. Dynamic regulation of middle cerebral artery blood flow velocity in aging and hypertension. Stroke. 2000;31:1897–903. 10926954 10.1161/01.str.31.8.1897

[39] Eames PJ, Blake MJ, Panerai RB, Potter JF. Cerebral autoregulation indices are unimpaired by hypertension in middle aged and older people*. Am J Hypertens. 2003;16:746–53. 12944033 10.1016/s0895-7061(03)00947-6

[40] Serrador JM, Sorond FA, Vyas M, Gagnon M, Iloputaife ID, Lipsitz LA. Cerebral pressure-flow relations in hypertensive elderly humans: transfer gain in different frequency domains. J Appl Physiol. 2005;98:151–9. 15361517 10.1152/japplphysiol.00471.2004

[41] Novak V, Novak P, Spies JM, Low PA. Autoregulation of cerebral blood flow in orthostatic hypotension. Stroke. 1998;29:104–11. 9445337 10.1161/01.str.29.1.104

[42] Sprint Research Group, Wright JT Jr., Williamson JD, Whelton PK, Snyder JK, Sink KM, et al. A randomized trial of intensive versus standard blood-pressure control. N Engl J Med. 2015;373:2103–16. 26551272 10.1056/NEJMoa1511939 PMC4689591

[43] Dolui S, Detre JA, Gaussoin SA, Herrick JS, Wang DJJ, Tamura MK, et al. Association of intensive vs standard blood pressure control with cerebral blood flow: secondary analysis of the SPRINT MIND randomized clinical trial. JAMA Neurol. 2022;79:380–9. 35254390 10.1001/jamaneurol.2022.0074 PMC8902686

[44] Newman L, O’Connor JD, Nolan H, Reilly RB, Kenny RA. Age and sex related differences in orthostatic cerebral oxygenation: findings from 2764 older adults in the Irish Longitudinal Study on Ageing (TILDA). Exp Gerontol. 2022;167:111903. 35902001 10.1016/j.exger.2022.111903

[45] Newman L, O’Connor JD, Romero-Ortuno R, Reilly RB, Kenny RA. Supine hypertension is associated with an impaired cerebral oxygenation response to orthostasis: finding from the Irish longitudinal study on ageing. Hypertension. 2021;78:210–9. 34058851 10.1161/HYPERTENSIONAHA.121.17111

[46] Zhang R, Witkowski S, Fu Q, Claassen JA, Levine BD. Cerebral hemodynamics after short- and long-term reduction in blood pressure in mild and moderate hypertension. Hypertension. 2007;49:1149–55. 17353511 10.1161/HYPERTENSIONAHA.106.084939

[47] Murphy CM, Kearney PM, Shelley EB, Fahey T, Dooley C, Kenny RA. Hypertension prevalence, awareness, treatment and control in the over 50s in Ireland: evidence from the irish longitudinal study on ageing. J Public Health. 2016;38:450–8. 10.1093/pubmed/fdv057 25922371

