# Supplementary material for: Orthostatic hypotension without co-existent supine hypertension is associated with impaired cerebral oxygenation: findings from the Irish Longitudinal Study on Ageing (TILDA)
Source: J Hum Hypertens. 2026 Apr 25;40(6):481–8. doi: 10.1038/s41371-026-01125-w (PMC13249562; doi:10.1038/s41371-026-01125-w)

Online Data Supplement

Orthostatic hypotension without co-existent supine hypertension is associated with impaired cerebral oxygenation: Findings from the Irish Longitudinal Study on Ageing (TILDA)

Louise Newman, John D. O'Connor, Richard B. Reilly, Rose Anne Kenny

Supplementary Table 1 – Participant characteristic by supine hypertension and orthostatic hypotension status, with the orthostatic hypotension thresholds adjusted for the presence of supine hypertension. Total sample N =2761.

	**No supine hypertension**		**Supine hypertension**		**Statistical test^ p-value**
	**No OH N 1266**	**Has OH N 134**	**No OH N 1273**	**Has OH N 88**	
Age (years), mean (SD)	63.2 (6.7)	67.8 (8.0)	66.0 (7.5)	70.1 (8.2)	<0.001*
Sex (female), n (%)	596 (47)	56 (42)	745 (59)	48 (55)	<0.001*
Highest educational attainment					
Primary/none, n (%)	187 (15)	29 (22)	222 (17)	20 (23)	
Secondary, n (%)	514 (41)	54 (40)	511 (40)	34 (39)	0.160
Tertiary, n (%)	565 (45)	51 (38)	540 (42)	34 (39)	
Height (cm), mean (SD)	167.6 (9.1)	169.0 (9.2)	164.8 (9.0)	165.7 (9.3)	<0.001*
Seated SBP (mmHg), mean (SD)	124.8 (15.0)	124.0 (16.1)	141.1 (17.2)	143.0 (18.7)	<0.001*
Seated DBP (mmHg), mean (SD)	77.5 (8.8)	74.9 (8.2)	84.8 (10.3)	832.1 (11.1)	<0.001*
Seated heart rate (bpm), mean (SD)	68.5 (11.2)	67.1 (11.1)	68.5 (10.7)	68.4 (10.7)	0.991
Supine SBP (mmHg), mean (SD)	124.9 (11.0)	125.9 (10.7)	157.6 (12.2)	167.2 (21.3)	<0.001*
Supine DBP (mmHg), mean (SD)	70.6 (7.1)	71.9 (6.9)	81.5 (9.0)	84.0 (12.0)	<0.001*
Supine heart rate (bpm), mean (SD)	65.0 (10.0)	64.6 (9.5)	65.6 (9.5)	65.8 (0.13)	0.084
Supine TSI (%), mean (SD)	72.7 (4.7)	72.2 (4.9)	72.7 (5.1)	72.6 (5.3)	0.670
PWV (m/sec), mean (SD)	10.0 (1.9)	10.9 (2.2)	10.8 (2.0)	11.8 (2.3)	<0.001*
BMI, mean (SD)	28.5 (4.8)	27.9 (4.8)	28.3 (4.4)	27.5 (5.07)	0.122
Smoking history					
Never	612 (48)	55 (41)	611 (48)	48 (55)	
Past	530 (42)	64 (48)	551 (43)	31 (35)	0.447
Current	124 (10)	15 (11)	111 (9)	19 (10)	
Alcohol excess, n (%)					
No (CAGE <2)	980 (77)	100 (75)	980 (77)	72 (82)	
Yes (CAGE>=2)	156 (12)	12 (9)	162 (13)	11 (12)	0.215
Did not respond	130 (10)	22 (16)	131 (10)	5 (6)	
Diabetes, n (%)	88 (7)	15 (11)	70 (5)	11 (12)	0.007*
CVD conditions					
= 0, n (%)	560 (44)	48 (36)	459 (36)	20 (23)	
= 1, n (%)	452 (36)	45 (33)	471 (37)	34 (38)	<0.001*

	No supine hypertension		Supine hypertension		Statistical test^ p-value
	No OH	Has OH	No OH	Has OH	
>= 2, n (%)	254 (20)	41 (31)	343 (27)	34 (39)	
Cancer, n (%)	34 (3)	7 (5)	39 (3)	2 (2)	0.407
Kidney disease, n (%)	6 (1)	1 (1)	6 (1)	1 (1)	0.825
Taking antihypertensive medications, n (%)	417 (33)	67 (50)	498 (39)	51 (58)	<0.001*
Taking antidepressant medications, n (%)	93 (7)	14 (10)	82 (6)	5 (6)	0.320
Taking antipsychotic medications, n (%)	11 (1)	4 (3)	18 (1)	1 (1)	0.164
Taking benzodiazepines, n (%)	11 (1)	2 (1)	16 (1)	1 (1)	0.775
CES-D score (max = 22), mean (SD)	3.8 (3.7)	3.6 (3.8)	3.8 (3.6)	4.5 (3.6)	0.501
TUG (s), mean (SD)	8.9 (2.2)	9.3 (2.2)	9.0 (1.8)	9.7 (2.03)	0.001*
Stand time transition (s), mean (SD)	7.2 (2.8)	7.6 (2.9)	7.3 (2.7)	8.4 (4.2)	0.123
MOCA, median (IQR)	27 (25 – 29)	26 (23 – 28)	26 (24 – 28)	26 (23 – 28)	<0.001*

Supplementary Figure 1 – Diastolic blood pressure response, adjusted for age, sex, education and height, conditional means and 95% confidence intervals from mixed-effects models. Absolute values (left) and change from baseline (right) are shown. SH – supine hypertension, OH40 – OH at 40 seconds post standing. Supine baseline level denoted by black dashed line.

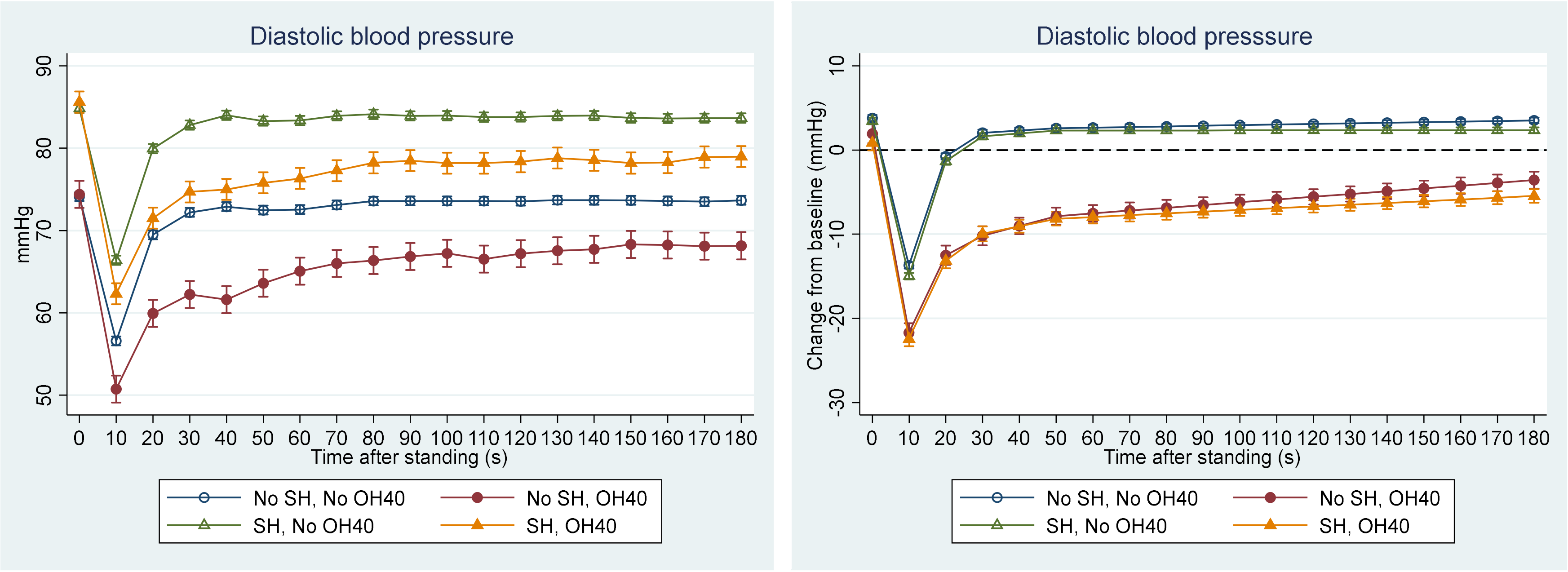

Supplementary Figure 2 – Heart rate response, adjusted for age, sex, education and height, conditional means and 95% confidence intervals from mixed-effects models. Absolute values (left) and change from baseline (right) are shown. SH – supine hypertension, OH40 – OH at 40 seconds post standing. Supine baseline level denoted by black dashed line.

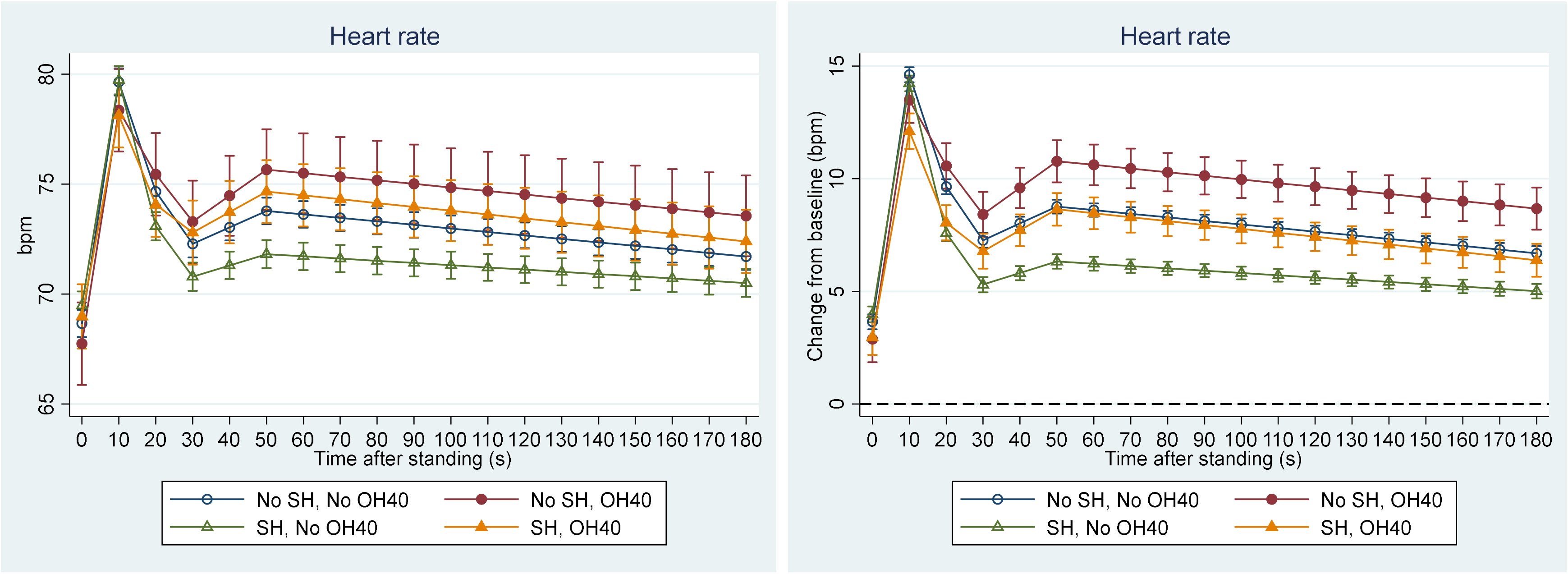

Supplementary Figure 3 – Systolic blood pressure response, adjusted for age, sex, education and height, conditional means and 95% confidence intervals from mixed-effects models. *OH40 BP thresholds have been adjusted for the presence of SH.* Absolute values (left) and change from baseline (right) are shown. SH – supine hypertension, OH40 – OH at 40 seconds post standing. Supine baseline level denoted by black dashed line.

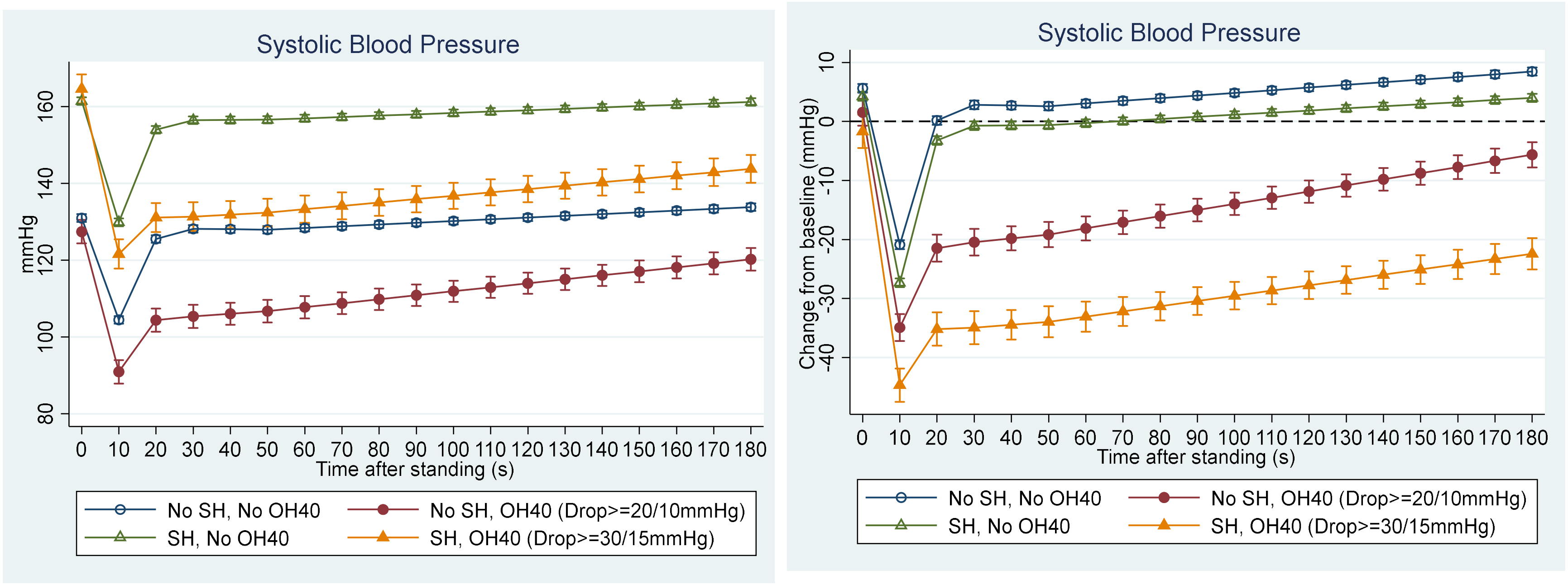

Supplementary Figure 4 – **Diastolic blood pressure response, adjusted for age, sex, education and height, conditional means and 95% confidence intervals from mixed-effects models.** ***OH40 BP thresholds have been adjusted for the presence of SH.*** Absolute values (left) and change from baseline (right) are shown. SH – supine hypertension, OH40 – OH at 40 seconds post standing. Supine baseline level denoted by black dashed line.

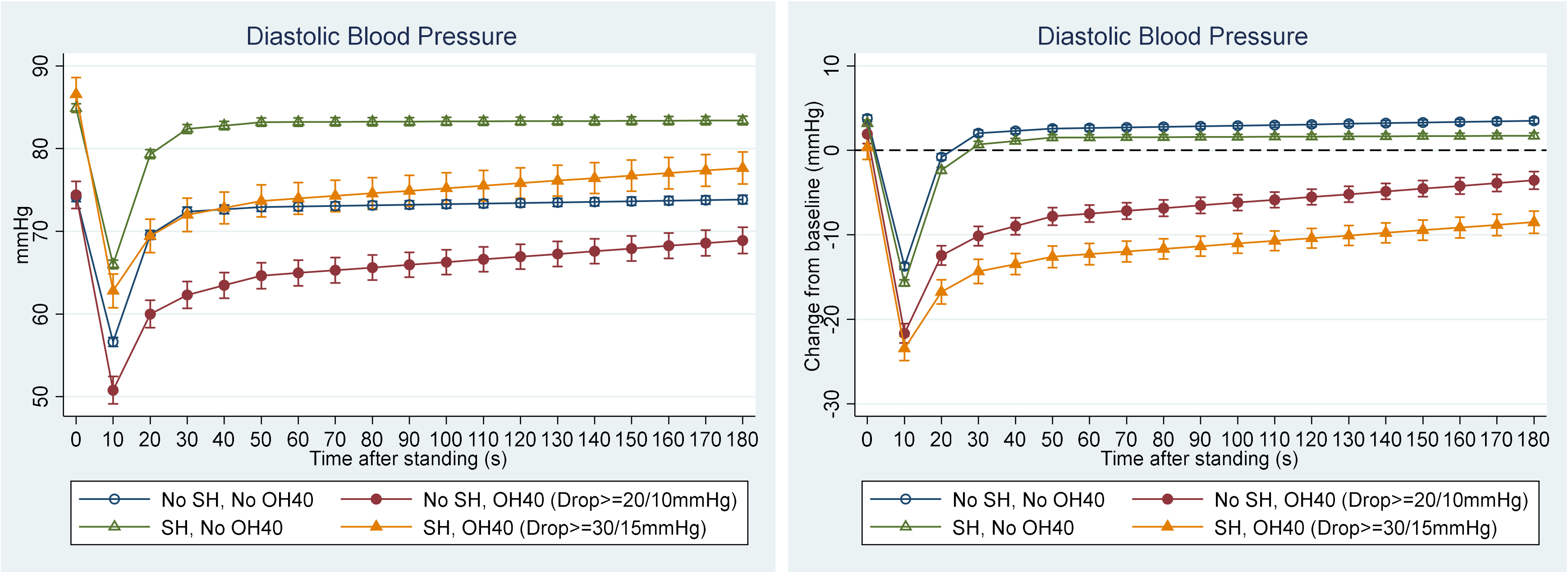

Supplementary Figure 5 – Heart rate response, adjusted for age, sex, education and height, conditional means and 95% confidence intervals from mixed-effects models. *OH40 BP thresholds have been adjusted for the presence of SH.* Absolute values (left) and change from baseline (right) are shown. SH – supine hypertension, OH40 – OH at 40 seconds post standing. Supine baseline level denoted by black dashed line.

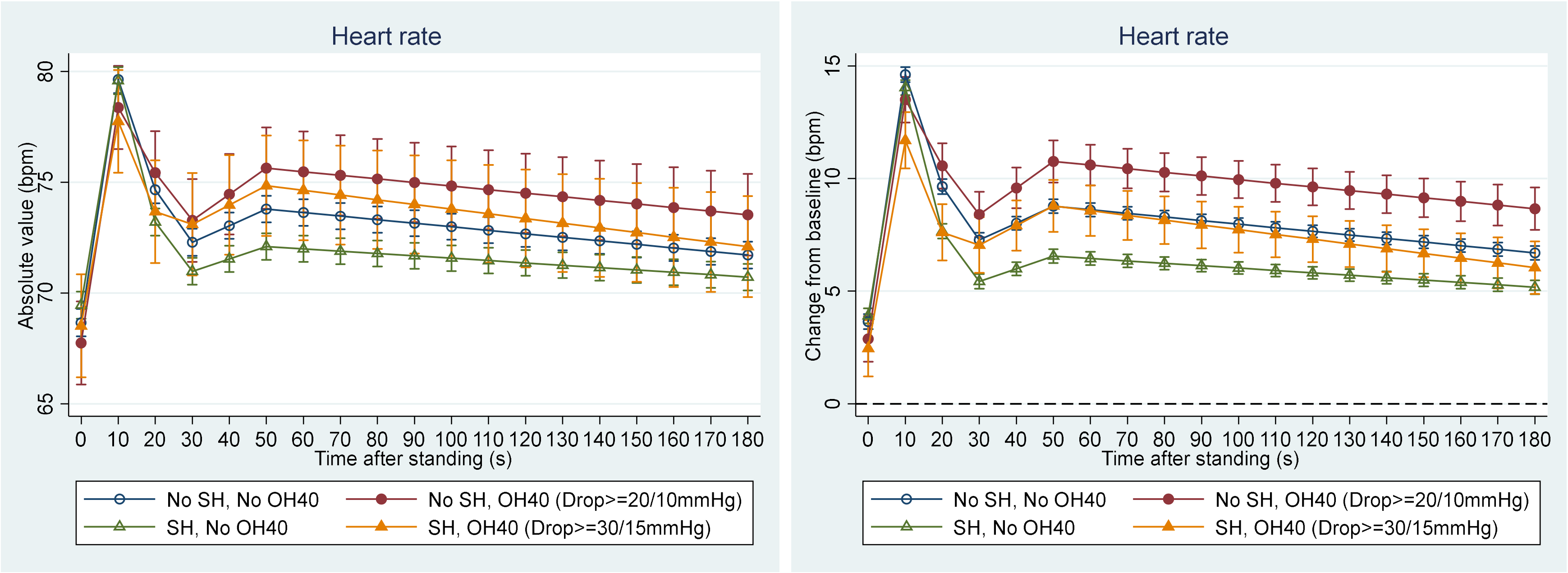

Supplementary Figure 6 – Tissue saturation index response, conditional means with 95% confidence intervals from univariate mixed-effects models. ***OH40 BP thresholds have been adjusted for the presence of SH.*** Absolute values (left) and change from baseline (right) are shown. SH – supine hypertension, OH40 – OH at 40 seconds post standing. Supine baseline level denoted by black dashed line.

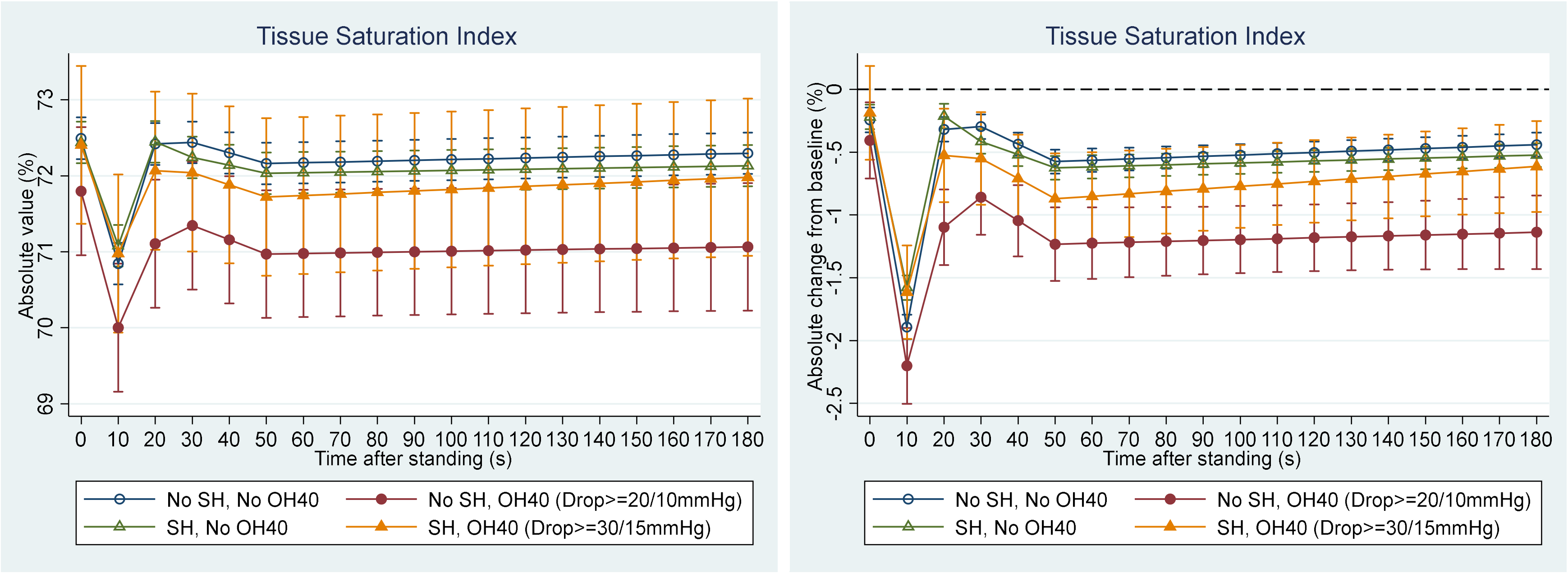

Supplement: Supplementary file 1 — Supplemental data [file 41371_2026_1125_MOESM1_ESM.pdf]
